# Enhancing Cochlear Implant Outcomes across Age Groups: The Interplay of Forward Focus and Advanced Combination Encoder Coding Strategies in Noisy Conditions

**DOI:** 10.3390/jcm13051399

**Published:** 2024-02-28

**Authors:** Telse M. Wagner, Luise Wagner, Stefan K. Plontke, Torsten Rahne

**Affiliations:** Department of Otorhinolaryngology, University Medicine Halle, Ernst-Grube-Straße 40, 06120 Halle (Saale), Germany; luise.wagner@uk-halle.de (L.W.); stefan.plontke@uk-halle.de (S.K.P.); torsten.rahne@uk-halle.de (T.R.)

**Keywords:** age-related hearing loss, cochlear implant, ForwardFocus, hearing effort, hearing in noise, speech recognition

## Abstract

**Background:** Hearing in noise is challenging for cochlear implant users and requires significant listening effort. This study investigated the influence of ForwardFocus and number of maxima of the Advanced Combination Encoder (ACE) strategy, as well as age, on speech recognition threshold and listening effort in noise. **Methods:** A total of 33 cochlear implant recipients were included (age ≤ 40 years: *n* = 15, >40 years: *n* = 18). The Oldenburg Sentence Test was used to measure 50% speech recognition thresholds (SRT50) in fluctuating and stationary noise. Speech was presented frontally, while three frontal or rear noise sources were used, and the number of ACE maxima varied between 8 and 12. **Results:** ForwardFocus significantly improved the SRT50 when noise was presented from the back, independent of subject age. The use of 12 maxima further improved the SRT50 when ForwardFocus was activated and when noise and speech were presented frontally. Listening effort was significantly worse in the older age group compared to the younger age group and was reduced by ForwardFocus but not by increasing the number of ACE maxima. **Conclusion:** Forward Focus can improve speech recognition in noisy environments and reduce listening effort, especially in older cochlear implant users.

## 1. Introduction

Hearing is one of the most essential prerequisites for communication [1] and an important component of social interaction and quality of life [2,3]. Untreated hearing disorders can impair cognitive performance and even contribute to the development of dementia [4,5,6]. If conventional hearing aid treatment is insufficient or not indicated, cochlear implants (CIs) may be an alternative to facilitate hearing by bypassing the inner ear and stimulating the auditory nerve electrically [7]. CI-mediated hearing differs from normal hearing in quality and timbre [8] and also speech perception is still not comparable to normal hearing.

Besides speech recognition in quiet [9], cochlear implantation also aims for good speech recognition in noise [10,11,12]. Hey et al. [13], however, showed that CI users with high levels of open-set speech recognition in quiet may still have poor hearing in noise compared to normal-hearing listeners [14]. To overcome this deficit, noise reduction algorithms were developed to improve the signal-to-noise ratio (SNR) and thus hearing with a CI [15]. The benefit of noise reduction technologies was, however, reduced with a reduction in the number of competing speakers [14,16,17]. A typical situation is the so-called cocktail party setting [18]. Normal hearing listeners can focus their listening attention on one specific speaker, even if there are other conversations in their immediate surroundings. In contrast, CI users need special noise reduction technologies to focus on the speaking person. Wimmer et al. [19] showed that in those situations speech recognition and SNR can be improved by using directional microphones.

A relatively new CI sound-processing algorithm is ForwardFocus (FF). ForwardFocus attenuates sounds from behind while preserving sounds from the front, in order to increase speech recognition in noise by increasing the SNR. To help CI users in focusing on frontal speakers more than directional microphones can do, FF was first implemented in the audio processor of the Nucleus 7 system (Cochlear, Sydney, Australia) [14].

Hey et al. [14] showed a superiority of 5.8 dB SNR with FF in comparison to directional microphone technology if the noise was presented from behind (90°, 180° and 270°). The impact of FF on speech perception in frontal noise presentation has not been investigated in detail. Since fluctuating noise signals are considered to have high ecological validity [20,21], an effect of FF on speech perception in icra5, a noise signal developed for audiological testing resembling the sound of a single speaker with pauses of 250 ms [22], would be expected as well.

Another option to increase speech recognition in Nucleus CI users is the Advanced Combination Encoder (ACE) strategy, which selects only the channels with the highest spectral energy in each stimulation cycle (number of maxima). It has been shown that the ACE algorithm is sensitive to noise since the wrong maxima could be selected, especially in speech gaps [23]. Berg et al. [24], however, showed that the maxima selection strategy of ACE improved sentences in noise discrimination and spectral modulation detection compared to a 16-channel continuously interleaved samples (CIS) strategy. Using sixteen versus eight maxima led to a significantly better understanding of monosyllables and sentences in a 20-talker babble noise signal for presenting speech and noise from the front (S_0_N_0_).

While a decrease in hearing in general underlies a degradation of peripheral hearing and cognitive decline, it is still unclear how age affects hearing with CIs. So far, only a few studies have examined aging in CI users and no study has investigated aging effects for FF and ACE maxima conditions. Shew et al. [25] examined two groups of bimodal CI users aged below and above 65 years. No difference between the age groups was found when hearing in quiet was measured, but the addition of noise resulted in a disproportional decline for the older participants. As a possible explanation, Füllgrabe [26] found that temporal processing deteriorates with age, starting in early midlife. The first significant deficits were observed in normal-hearing participants between the ages of 40 and 49 years, even in the absence of peripheral hearing loss. Above the age of 40 years, the maximum word recognition score also starts to decline, as observed in a large cohort [27].

Since CI outcome depends on cognitive factors, listening effort, as measurement of these factors, has been introduced as an important assessment [28]. After cochlear implantation, the available acoustic and spectro-temporal cues are limited, which would cause an increased listening effort [9]. This assumption was recently confirmed by a review of 24 studies that showed higher levels of listening effort in CI users when compared to normal-hearing controls using scales, questionnaires, and electroencephalogram measurements [29]. Nevertheless, the main factors leading to this difference still need to be clarified. It is hypothesized that the technical parameters of cochlear implants greatly impact listening effort, more than age or cognitive factors do [9]. Other studies discussed the effects of cognitive factors such as working memory capacity or inhibitory control on listening effort [29,30] and found effects of aging on listening effort in CI users [31]. According to a study by Perrau et al., other variables, such as the duration of CI use and the age of onset of hearing loss, were not significantly related to listening effort [31].

This study aimed to evaluate the effects of FF and number of ACE maxima on speech recognition and listening effort in spatial conditions when fluctuating noise is presented from the front or the back. A younger group and an older group of CI users were included and compared to age-matched reference data of normal-hearing listeners [32]. We hypothesized that an increased number of ACE maxima would increase the spectral information of the CIs and therefore could reduce listening effort in noise and that the increased SNR by using FF would reduce listening effort in noise as well.

## 2. Materials and Methods

In a prospective, non-interventional exploratory cohort study, CI users between 18 and 80 years of age were included and allocated to the age groups of ≤40 years and >40 years. Inclusion criteria were a post-lingually acquired CI indication; use of a Nucleus CI24RE, CI5xx, or CI6xx device (Cochlear, Sydney, Australia) with at least 20 active electrode contacts; use of the ACE coding strategy for at least six months; being a German native speaker; and having a CI-aided monosyllabic word recognition score (WRS) in quiet of at least 50% at 65 dB SPL. This study was conducted in a laboratory of experimental audiology at a university hearing and implant center. This study was approved by the local ethical review board (approval number 2021-044) and conducted in compliance with the Declaration of Helsinki. Informed written consent was obtained from all participants.

All measurements were performed in a unilateral setting with the same Nucleus 7 audio processor. For bilaterally implanted CI users, the side with the better WRS was chosen. Each individual patient’s device fitting was used as base for the study settings: eight maxima without FF (M8/FF−), eight maxima with activated FF (M8/FF+), and twelve maxima with activated FF (M12/FF+). If the individual setting did not allow for twelve maxima, the largest possible number of maxima was used instead.

**Figure 1:**
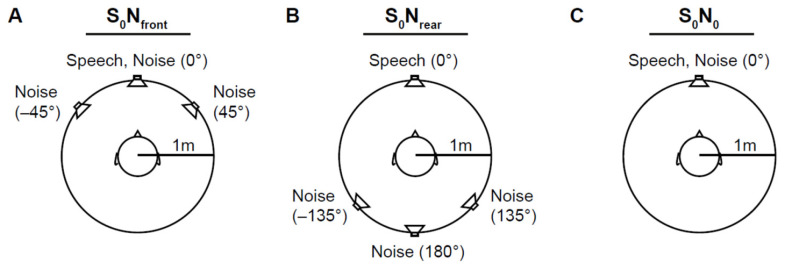
Experimental conditions with speech presented from the front (0°): (**A**) frontal noise condition with noise signals presented from 0°, +45°, and −45° (S_0_N_front_); (**B**) rear noise condition with noise signals presented from 180°, +135°, and −135° (S_0_N_rear_); and (**C**) S_0_N_0_ reference condition with only one frontal noise source (0°).

Participants were positioned in a circle at a distance of 1 m from the loudspeakers in a sound-attenuated room. The head was fixed with a papillon head fixation system. The German matrix sentence test OLSA (HörTech, Oldenburg, Germany) [9,33,34] was utilized to measure the 50% speech recognition threshold (SRT_50_) in noise with a constant sound pressure level of 65 dB. Figure 1 shows the three spatial configurations of speech and noise presentation. The speech signal (S) of the target speaker was always presented in front (0°, S_0_). Noise signals (N) were either the generic noise of the OLSA with a male voice (olnoise) or the icra5 fluctuating noise of the OLSA. In the frontal noise condition, the noise signals were presented from 0°, +45°, and −45° (S_0_N_front_). The rear noise presentation locations were 180°, +135°, and −135° (S_0_N_rear_). The S_0_N_0_ condition was used as the reference and training condition. Participants were presented with lists of 20 sentences with adaptive speech levels in continuous noise. Within the adaptive measurement of SRT_50_, after every sentence, the sound pressure level was adjusted based on the participant’s response to the preceding sentence. Two training lists, one for olnoise and one for icra5, were used before the 12 test runs started in a pseudorandom sequence. For the spatial conditions of S_0_N_front_ and S_0_N_rear_, the test runs comprised olnoise with M8/FF− and icra5 with M8/FF−, M8/FF+, and M12/FF+.

Listening effort was measured using the “Adaptive Categorical Listening Effort Scaling” (ACALES) test (Hörtech, Oldenburg, Germany) [9,35,36] in the S_0_N_0_ condition. The participants were asked to rate their listening effort on a scale of eight response categories ranging from ‘no effort’ to ‘only noise’ following the frontal presentation of two sentences from the OLSA test in 65 dB SPL icra5 noise. Based on the previous rating, the SNR was changed adaptively for every set of two sentences. SNR limits of −40 dB and +20 dB were applied. The SNR_cut_, which is the SNR at which a moderate effort (4 effort scale categorical units, ESCUs) was measured, was assessed for all experimental conditions.

The SRT_50_ and SNR_cut_ distributions were descriptively reported and analyzed. Normality was assessed through the Shapiro–Wilk test. To compare the distributions of SRT_50_ between the spatial configurations, the signal processing settings, and age, an ANOVA for repeated measures with the within-subject factors of ‘spatial condition’, ‘FF’, and ‘Maxima’ and the between-subject factor of ‘age group’ was used. Mauchly’s test was utilized to verify the assumption of sphericity and Greenhouse–Geisser correction was applied if necessary. The effect of the age group on the SNR_cut_ was analyzed with an ANOVA for all used experimental conditions. Bonferroni correction was applied to adjust the degrees of freedom for all post hoc comparisons. The required sample size was based on the SRT_50_ as the primary endpoint with an assumed standard deviation of 2 dB. To calculate the 95% confidence intervals with an assumed length of 2 dB, a sample size of 18 participants per age group resulted. This is comparable to studies that have already been successfully conducted with similar methodologies and research questions. The statistical analyses were performed using version 28 of the SPSS software from IBM in Ehningen, Germany. The level of significance was set to *p* = 0.05.

## 3. Results

Thirty-three participants were recruited. One participant with SRT_50_ values above 30 dB SNR was excluded from the analysis. Table 1 provides the demographic and implantation data of all analyzed participants. Electrode insertion was mostly through the round window, in four cases after a partial or subtotal cochleoectomy, and in two cases through a cochlestomy.

**Figure 2:**
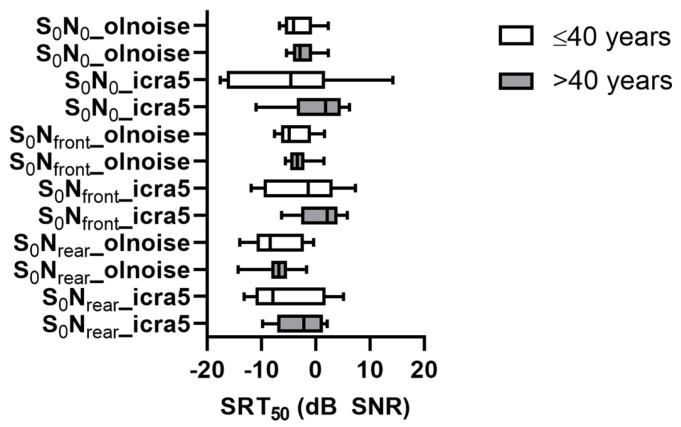
Speech recognition threshold (SRT_50_) for all spatial and noise conditions for the two age groups, ≤40 years (white) and >40 years (grey), presented as boxplots for all analyzed participants with deactivated FF and using 8 ACE maxima (M8/FF−). The whiskers show minimum and maximum values.

Figure 2 shows the SRT_50_ for all spatial and noise conditions for the two age groups. The respective mean SRT_50_ and standard deviations are shown in Table 2. The ANOVA of SRT_50_ shows no effect of the age group (F(1,30) = 2.3; *p* = 0.142). The effects of spatial condition (F(1.5,45.3) = 35.5; *p* < 0.001), noise (F(1,30) = 7.6; *p* = 0.010), and the interaction of spatial condition and noise (F(1.5,45.5) = 11.7; *p* < 0.001) were significant. Post hoc comparisons show better SRT_50_ for olnoise (−4.53 dB SNR) as compared to icra5 (−2.45 dB SNR). The SRT_50_ for S_0_N_rear_ (−5.69 dB SNR) was significantly better than for S_0_N_0_ (−2.63 dB SNR), all other differences were not significant. Since age group had no effect on the SRT_50_ results, both groups were combined for further analysis.

**Figure 3:**
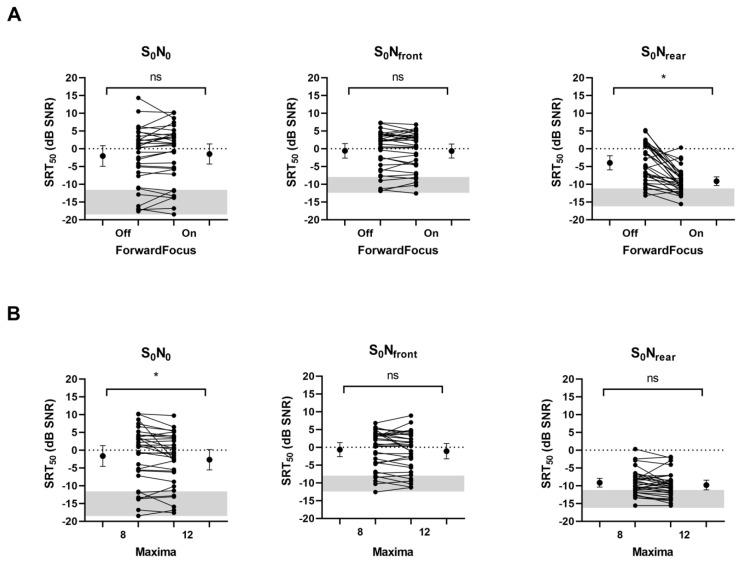
(**A**) Effect of ForwardFocus and (**B**) number of ACE maxima on the speech recognition threshold in noise (SRT_50_) for different spatial conditions of signal and noise presentations. Individual data are presented together with the means ± SD. Significant differences are marked with an asterisk (*p* < 0.05); ns: not significant. The reference data for normal hearing listeners [32] are displayed as grey bars (means ± SD).

The effect of FF on the SRT_50_ is shown in Figure 3, together with reference data of normal-hearing listeners [32]. FF improved the SRT_50_ in the S_0_N_rear_ condition from −4.0 dB SNR (SD: 5.5) to −9.1 dB SNR (SD: 3.4) (*p* < 0.001), but not in the other spatial conditions.

The effect of an increased number of ACE maxima is also shown in Figure 3. The missing values of two participants were imputed based on the respective mean SRT_50_ of the age group (S_0_N_0_: −2.69 dB, S_0_N_front_: −1.09 dB, S_0_N_rear_: −9.81 dB). The increased number of ACE maxima improved the SRT_50_ in the S_0_N_0_ condition from −1.6 dB SNR (SD: 8.1) to −2.7 dB SNR (SD: 7.4) (*p* = 0.033), but not in the other spatial conditions.

All participants participated in the ACALES test. The test was terminated if the SNR of the stimulus presentation exceeded 16 dB. This affected four participants in the younger age group and six participants in the older age group. In addition, five more participants in the older age group could not complete the test for only one or two of the three conditions. In these cases, the missing values were replaced by a value of 10 dB SNR.

**Figure 4:**
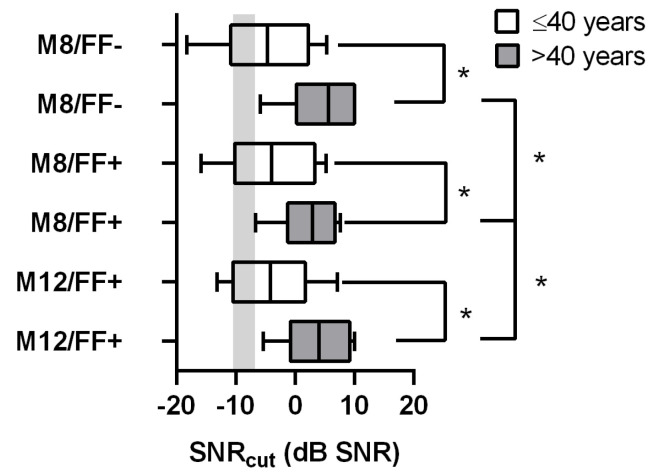
Listening effort in noise displayed as boxplots for different signal processing conditions and age groups. Significant differences are marked with an asterisk (*p* < 0.05). The reference data for normal hearing listeners [32] are displayed as grey bars (means ± SD).

Figure 4 shows the SNR_cut_ results of the ACALES test for listening effort. The mean values are reported in Table 2. The ANOVA of SNR_cut_ shows an effect of age group in the M8/FF− condition (F(1,21) = 11.5; *p* = 0.003), the M8/FF+ condition (F(1,21) = 6.7; *p* = 0.017), and the M12/FF+ condition (F(1,21) = 7.6; *p* = 0.012). In the older age group, SNRcut showed a significant increase of 9.5 dB for M8/FF−, 6.6 dB for M8/FF+, and 7.2 dB for M12/FF+. In the younger group, no significant differences in SNR_cut_ were measured between the FF and ACE maxima conditions. FF significantly improved the SNR_cut_ by 2.4 dB (*p* = 0.027) in the older age group; however, the additional increase in ACE maxima worsened it by 1.2 dB (*p* = 0.0495).

## 4. Discussion

The results show that FF improves speech recognition in noise when fluctuating noise is presented from rear directions and has no effect on SRT if noise is frontally presented. Since noise sources from behind often occur in daily life, this result is relevant for CI users. However, not all CI users benefited from activated FF, and 4 of the 32 analyzed participants even achieved worse results. Possible reasons for this are that two of these four participants reported very short daily CI wearing times (1 h/d) and were potentially not used to hearing with the CI in general. For another participant, we suspect that cognitive factors interacted with performance in our study.

Apparently, there is a slight trend that participants with poor speech recognition in noise profited more from FF than participants with good speech recognition in noise. An analysis of two previous studies [14,37] indicates similar characteristics in the corresponding patient cohorts. However, this needs to be confirmed in a dedicated future investigation. Overall, the FF-induced improvement in speech recognition in noise for the S_0_N_rear_ condition is in line with the results of Hey et al. for the S_0_N_90,180,270_ condition [14]. The present study complements those findings by showing similar results with the Nucleus 7 audio processor. As expected from the signal processing design of FF [14], speech recognition in noise could not be improved if noise came from the front (S_0_N_front_, S_0_N_0_).

A limitation of this unilateral study design is that in bilaterally implanted CI users, FF was only activated in one CI. Those participants reported a somehow unbalanced hearing and would potentially benefit even more from FF being activated in both audio processors. Also, the duration of deafness before CI implantation and CI experience could influence speech recognition in noise. To avoid a resulting bias, both age groups were balanced according to CI experience.

When the number of ACE maxima was increased to 12, the results show significant SRT_50_ improvements only for the S_0_N_0_ condition. We hypothesize that in less complex listening situations, such as the S_0_N_0_ condition, spatial cues cannot be utilized to separate speech and noise. An increased number of maxima would then potentially provide more speech-related information, which helps to better discriminate speech from noise. If noise comes from three directions (S_0_N_front_ or S_0_N_rear_) and speech only from one, ACE maxima selection would also pick up signals from all three directions based on their amplitudes and increase the difficulty of discriminating the speech signal from noise based on spatial information alone.

These results are in line with those of Berg et al. [24], who showed significant improvements using 16 versus 8 maxima in the ACE strategy for sentence recognition in noise. The S_0_N_0_ condition is suitable for standardized audiometry but with questionable impact for everyday hearing. We assume that FF and increased ACE maxima could be used simultaneously to achieve better speech recognition in such less-complex situations. Nevertheless, for difficult listening conditions (S_0_N_rear_), which often appear in real-life settings, the results show that increasing the number of maxima from 8 to 12 does not provide significant benefits over FF in speech perception overall. In the present study, however, some participants could only be measured with 9 to 11 maxima instead of 12, and other parameters such as electrode-to-modiolus distance depending on the type of electrode carrier used also varied between participants. We recommend future studies to investigate the number of ACE maxima independently of FF in different spatial conditions. Also, in our data, we could see some that patients improved their results and could observe a trend that the best mean value for speech perception (−9.8 dB), whenever not significant, was found in the S_0_N_rear_ condition using 12 maxima.

In our study, age group did not affect speech recognition thresholds in noise. In contrast to a study by Füllgrabe et al. [26], which described a decreased processing of temporal fine structure cues with age, starting between 40 and 49 years, even without peripheral hearing loss in normal hearing listeners, we did not find a deterioration in SRT_50_ in fluctuating noise with age. Also contrasting with our results, Shew et al. [25] demonstrated that the addition of noise disproportionally affects the speech recognition of adults over 65 years old. Either age did not have a significant impact in our test setting at all or the detrimental effect of age has a later onset than reflected in our age groups, which had a cutoff at 40 years, or even neither is true. The time of implantation and daily wearing time likely affect hearing in CI users more than age does [38,39].

Compared with age-specific reference data for the same spatial signal and noise configurations [32], the present results demonstrate that some CI users are able to perform similar to normal-hearing listeners. Thus, despite the fact that studies often overestimate the performance of CIs due to the experimental design [40,41], we conclude that FF provides a tool that significantly reduces hearing deficits in some situations.

Compared to olnoise, the results show poorer SRT_50_ for fluctuating icra5 noise. This confirms that fluctuating competing signals are more difficult for CI users. [7,14,42] In the same experimental design as in the present study, normal-hearing listeners showed better SRT_50_ in icra5 noise compared to olnoise. While normal-hearing people can benefit from the so-called gap listening [43], CI users could possibly not detect the speech signals in the pauses of only 250 ms within the icra5 noise signal. This is consistent with a study by Rader et al. [44], who were also unable to detect gap listening in CI users in modulated noise.

Although hearing tests in noise are becoming more relevant in everyday clinical practice, their ecological validity needs to be improved [41]. Several studies have shown that fluctuating interfering signals or competing talkers [45] can best reflect everyday situations [42,46]. Hey et al. [41] recently revealed that a stationary noise cannot replace interfering signals if used in speech audiometry. The results of our study are based on the recommended icra5 noise [22,47] and can thus contribute to the standardization of clinical measurements, especially in comparison with the recently published data of normal-hearing subjects [32].

In contrast to the results for speech intelligibility in noise, we found a significant age dependency in listening effort across all experimental conditions. Older CI users (>40 years) showed a larger listening effort in noise than those in the age group ≤40 years. Although speech recognition measurements were possible in these participants, some in the group >40 years could not even complete the ACALES test because even the largest possible SNR was rated as too exhausting. This was not observed in the younger age group. The differences between the age groups might result from the deterioration in temporal fine structure processing with age [26] or other cognitive factors like processing speed, executive control, and working memory capacity, which exhibit known age effects [9,30].

We found that FF reduced listening effort only in the group >40 years. From this, we conclude that reducing background noise might reduce cognitive effort and thus listening effort. This finding might be explained by the “Framework for Understanding Effortful Listening” (FUEL) model, which describes listening effort as a multifactorial construct, consisting of cognitive, motivational, and input-related demands. It suggests that speech recognition and listening effort can differ due to individual cognitive factors [9,48]. We assume that the input-related benefits of the FF microphone technology decreased the cognitive demand for speech comprehension, which is considered to be higher in older listeners [49], and led to reduced listening effort in that group. Based on this theory, younger CI users might have more cognitive resources, e.g., better temporal processing, that make listening effort more independent from input-related speech recognition. However, since this study was powered for speech perception in noise, future studies should address the effects of age on listening effort in noise in more detail.

Additionally, FF reduced listening effort for older CI users in the S_0_N_0_ condition, for which no improvements in speech recognition could be expected. This confirms the theory that speech recognition and listening effort are not aligned, and FF can improve listening effort independently from an improvement in speech recognition.

Another limitation of this study is that some participants were experienced FF users and others activated it for the first time, which could have introduced bias to the results. While data on this subject are very limited, it is being discussed that experienced FF users may have advantages in speech recognition, also in noise, due to training effects comparable to an acclimatization effect of new coding strategies [50].

It was also observed that most CI users had a markedly higher listening effort than normal-hearing listeners, which was reflected in the relatively high dropout rates in the ACALES test. We suggest that future investigations include more spatial conditions, investigating the relation between improved speech recognition scores as for the S_0_N_rear_ condition and reduced listening effort, including training with FF to examine this observation more concisely.

Age-mediated cognitive factors or other, not examined, factors linked with auditive processing, such as age at the time of implantation, which can be associated with decreased neuronal plasticity or the time of deafness before implantation, could explain the observed effects of age on listening effort. Our groups, however, were not matched with respect to these factors. Age-dependent listening effort based on cognitive changes should also have affected the normal-hearing reference group of Rahne et al. [32]. However, these data show an age effect only on speech recognition in noise but not on listening effort. This might be explained by the finding of Abdel-Latif and Meister [9] showing that the outcomes of cognitive tests (processing speed, executive control, and working memory capacity) were correlated with age, but no impact of age and cognitive abilities on listening effort in CI users was found. The authors concluded that listening effort was dominated by device-related, technical factors. The results of our study suggest that FF decreases listening effort independently from speech recognition. As a consequence, the assessment of listening effort should be included into clinical routine assessment after CI provision.

To summarize, we conclude that FF can significantly improve speech recognition in the presence of multiple noise sources presented from the rear, which can improve hearing in daily life. ForwardFocus reduced listening effort in older CI users, for whom the listening effort is larger compared to younger and normal-hearing listeners. The number of ACE maxima decreased the speech recognition threshold in noise if speech and noise were frontally presented and did not affect listening effort. Generally, speech recognition in noise was not found to be age-dependent. However, future studies should focus on individual effects of aging and age-related cognitive effects on listening effort in CI users.

## Figures and Tables

**Table 1:** Anamnestic data and baseline characteristics.

	Age Groups		
Characteristics	≤40 Years	>40 Years	All
Number	14	18	32
Age, mean (SD), years	28.6 (7.9)	70.0 (7.2)	51.9 (22.1)
Median [25th, 75th percentiles]	27.0 [20.0, 36.3]	69.5 [62.8, 76.3]	61.0 [29.5, 71.5]
Men/women, N	9/5	13/5	22/10
Right/left CI, N	5/9	8/10	13/19
Word recognition, mean (SD), % correct at 65 dB SPL			
Ipsilateral	71 (17)	73 (15)	72 (15)
Median [25th, 75th percentiles]	73 [58, 85]	75 [60, 85]	75 [60, 85]
Contralateral	65 (40)	59 (23)	62 (31)
Median [25th, 75th percentiles]	78 [34, 100]	65 [53, 75]	68 [48, 84]
Active electrodes, mean (SD), *n*	21.8 (0.8)	21.8 (0.5)	21.8 (0.6)
Median [25th, 75th percentiles]	22.0 [22.0, 22.0]	22.0 [22.0, 22.0]	22.0 [22.0, 22.0]
Pulse width, mean (SD), ms	35.5 (13.7)	29.7 (7.5)	32.3 (10.9)
Median [25th, 75th percentiles]	37.0 [25.0, 37.0]	25.0 [25.0, 37.0]	25.0 [25.0, 37.0]
Stimulation rate, mean (SD), Hz	921.4 (80.2)	977.8 (186.5)	953.1 (150.2)
Median [25th, 75th percentiles]	900.0 [900.0, 900.0]	900.0 [900.0, 1200.0]	900.0 [900.0, 900.0]
CI usage per day, mean (SD), hours	11.5 (5.5)	13.7 (2.3)	12.8 (4.1)
Median [25th, 75th percentiles]	13.0 [8.8, 15.3]	14.0 [12.0, 15.2]	14.0 [11.3, 15.0]
CI experience, mean (SD), years	5.9 (4.9)	5.1 (3.2)	5.4 (4.0)
Median [25th, 75th percentiles]	4.5 [1.7, 9.5]	4.0 [2.8, 7.0]	4.0 [2.0, 9.0]

**Table 2:** Speech recognition and listening effort in noise outcomes.

Age Group, Years			Age Group, Years		
Outcome			≤40	>40	Total
Speech recognition in noise					
SRT_50_ in dB SNR, mean (SD)					
	olnoise M8/FF−	S_0_N_0_	−3.2 (2.9)	−2.3 (2.4)	−2.7 (2.6)
		S_0_N_front_	−3.9 (3.0)	−3.1 (1.9)	−3.5 (2.4)
		S_0_N_rear_	−7.5 (4.4)	−7.0 (2.8)	−7.2 (3.5)
	icra5 M8/FF−	S_0_N_0_	−5.1 (10.6)	0.1 (5.1)	−2.2 (8.2)
		S_0_N_front_	−2.6 (7.1)	1.0 (3.8)	−0.6 (5.7)
		S_0_N_rear_	−5.3 (6.8)	−2.9 (4.1)	−4.0 (5.5)
	icra5 M8/FF+	S_0_N_0_	−4.7 (9.9)	0.7 (5.5)	−1.6 (8.1)
		S_0_N_front_	−2.8 (6.6)	1.0 (3.7)	−0.7 (5.4)
		S_0_N_rear_	−9.7 (4.0)	−8.7 (2.9)	−9.1 (3.4)
	icra5 M12/FF+	S_0_N_0_	−5.9 (8.8)	−0.2 (5.0)	−2.7 (7.4)
		S_0_N_front_	−3.1 (6.6)	0.5 (4.1)	−1.1 (5.5)
		S_0_N_rear_	−10.3 (4.6)	−9.5 (2.4)	−9.8 (3.5)
Listening effort in noise					
SNR_cut_ in dB SNR (SD)					
	icra5 M8/FF−	S_0_N_0_	−4.8 (7.8)	4.7 (5.7)	0.1 (8.2)
	icra5 M8/FF+	S_0_N_0_	−4.3 (7.4)	2.3 (4.8)	−0.9 (6.9)
	icra5 M12/FF+	S_0_N_0_	−3.7 (7.0)	3.5 (5.4)	0.1 (7.1)

Abbreviations: SNR: signal-to-noise ratio; SD: standard deviation; SPL: sound pressure level; SRT50: 50% speech reception threshold; SNR_cut_: signal-to-noise ratio at moderate listening effort.

## Data Availability

Supporting raw data may be obtained through special request from the corresponding author.
